# Causal machine learning for extracting insights from observational radiotherapy data

**DOI:** 10.1038/s41746-026-03214-z

**Published:** 2026-09-14

**Authors:** Margaret Y. Sui, Kyra L. Rosen, Ariel Yuhan Ong, Joseph C. Kvedar

**Affiliations:** 1 Harvard Medical School, Boston, MA USA; 2 Massachusetts Institute of Technology, Cambridge, MA USA; 3 Broad Institute, Cambridge, MA USA; 4 Institute of Ophthalmology, University College London, London, UK; 5 Moorfields Eye Hospital NHS Foundation Trust, London, UK; 6 NIHR Moorfields Biomedical Research Centre, London, UK

**Keywords:** Cancer, Computational biology and bioinformatics, Oncology

## Abstract

While clinical trials are the gold standard for determining causative side effects from treatment, some trials are too logistically or ethically challenging to complete. Many side effects of treatments are learned from retrospective analyses; however, these can be confounded by other random variables. Causal and explainable machine learning tools are promising for teasing apart confounders from real treatment side effects in observational data.

## Introduction

Determining true side effects of treatment can be challenging because many side effects can take years to develop or be rare enough that they require a large clinical cohort to detect^1–3^. Thus, while clinical trials would be the most rigorous way to quantify such side effects, the length of study and resources required to detect some side effects might be prohibitively expensive^4,5^. Many treatment side effects end up being uncovered correlationally from retrospective studies. But while retrospective studies are helpful for suggesting potential side effects, without a controlled study, it can often be hard to determine if the side effects are truly caused by the treatment itself^2,3,6^.

Recent advances in causal machine learning allow researchers to estimate side effects from observational data by adjusting for confounding variables^7^. One approach is generalized random forests (GRF)^8–10^, which stratify a study population by different characteristics, such as age or sex, and measure whether the split separates participants into those who are more or less likely to have the side effect. Different ways of splitting the patient population are tested randomly to see which ones help explain variation in the expected treatment side effects the most. By determining if specific characteristics like being of a certain age affect the side effect outcomes, one can determine which populations to be most cautious about monitoring.

Radiation oncology is an excellent field to demonstrate the efficacy of causal machine learning because nearly all radiotherapy outcome studies to date have been observational^7^. This is due to the lack of funding for clinical trials on radiation therapy because vendor profit is only from one-time selling of the initial devices, unlike pharmaceuticals^11,12^. Additionally, similar to surgeries, radiation is a procedure that makes placebo comparators difficult to execute^13^. Without many clinical trials, practitioners rely on the findings from observational studies. However, observational studies often cannot be used to definitively guide dose adjustments because many confounders exist in the data^14–18^. For instance, while radiation is known to cause mandibular osteoradionecrosis, a dangerous side effect in which the jaw bone dies due to damaged blood supply, the impact is often confounded by dental disease, chemotherapy, smoking, and diabetes^17–20^. In order to tease apart the extent to which osteoradionecrosis development depends on specifically radiation dosage versus other confounding factors, Chen et al. used GRFs on a dataset with 931 head and neck cancer patients to estimate the impact of radiation dosage on osteoradionecrosis incidence^7^.

## Identifying age as a mediator for mandibular osteoradionecrosis

Interestingly, Chen et al. found that the most important factor that mediates whether osteoradionecrosis develops was age^7^. Those who were 50–60 years old were more likely to develop osteoradionecrosis following radiation treatment compared to those younger and older. Chen et al. determined this by using explainable machine learning tools including the Shapley additive explanations (SHAP) importance and the GRF split-based importance^7^. The SHAP importance quantifies how much a specific patient characteristic contributes to either increasing or decreasing the chances of an outcome^21^, in this case mandibular osteonecrosis. The GRF split-based importance measures how many times a specific clinical characteristic (i.e., age) is used as a split in different iterations of generated trees that partition patients into groups that are more or less likely to have the mandibular osteonecrosis side effect^10^. Overall, both the GRF-split and SHAP importance help explain how much specific patient characteristics contribute to the mandibular osteonecrosis side effect. In the Chen et al. study, the GRF-split based importance and SHAPs value were 44% and 39% respectively for age, as opposed to <20% for all other factors like tumor stage, smoking status, hypertension, diabetes, chemotherapy and dental extraction, suggesting that age within 50–60 years old is the strongest predictor of mandibular osteonecrosis. Interestingly, diabetes status had the lowest SHAP and GRF-split based importance metrics at only 2% each, making it the weakest predictor of mandibular osteoradionecrosis post-radiation^7^.

It is important to note that while a high importance value suggests strong association between age and mandibular osteoradionecrosis post-radiation, it does not establish causation. Metrics like SHAP values and GRF-split based importance values help explain how much specific characteristics contribute to the osteoradionecrosis incidences seen, but they do not make causal predictions of what would have happened had the patients not undergone radiation or undergone less radiation. To causally determine if age impacted osteoradionecrosis incidence, the authors calculated the conditional average treatment effect (CATE)^8,10^, which predicts how much the treatment effect changes for a single unit increase in radiation in specific age subgroups. With CATE, the authors again found that the treatment effect from radiation was largest for those in the 50–60 age group^7^.

While metrics like CATE and causal inference tools are helpful for interpreting observational data, it is important to remember that these calculations make several assumptions. One is accountability, which assumes that all variables that influence either radiation dose exposure or osteoradionecrosis outcome have been measured and adjusted for. While the authors covered many variables, unmeasured factors like bone density, nutritional status, genetic susceptibility, and socioeconomic determinants of post-treatment care were not captured in this data^7^. Thus, while these causal inferences from observational data serve as a useful guide for informing future clinical trials, they still benefit from corroborating clinical trials.

## Role for machine learning in clinical medicine

One vision for the future would be to have these explanatory and causal machine learning tools train on large amounts of amassed observational data from many different centers to predict optimal treatment doses that can then be tested in targeted clinical trials. This approach could turn observational data into a more useful clinical tool. Currently, there are many cancers such as childhood leukemias that may be overtreated to maximize chance of cure. While we now achieve high rates of leukemia cure, many children live with lifelong side effects like heart damage, fertility struggles, or neurocognitive challenges^22^. Clinical trials are already being performed to test shorter durations of chemotherapy to see if high cure rates for some leukemias can be maintained even at lower doses. Chen et al. demonstrate that explanatory and causal machine learning approaches, such as GRFs and CATE, can help predict for which patients these clinical trials could be most safely done. For instance, if certain side effects happen most often in children of a certain age, perhaps dose-lowering trials should target children of that age.

## Conclusion

Causal and explainable machine learning serves as a promising frontier for improving personalized medicine and determining which patient cohort subgroups would most benefit from adjustments in treatment doses. Clinical trials can be long and arduous, so the more useful information there is to guide recruitment decisions for who to enter into these clinical trials, the more promising trial outcomes would be. Causal and explainable machine learning tools could also be used on incoming preliminary data from adaptive clinical trials to guide future patient selection in the trial. With better trial design and implementation, improvements in clinical care guidelines could result in better medical outcomes for patients.

## Data Availability

No datasets were generated or analysed during the current study.
